# The role of infected epithelial cells in *Chlamydia*-associated fibrosis

**DOI:** 10.3389/fcimb.2023.1208302

**Published:** 2023-05-17

**Authors:** Liam T. Caven, Rey A. Carabeo

**Affiliations:** ^1^ Department of Pathology and Microbiology, University of Nebraska Medical Center, Omaha, NE, United States; ^2^ School of Molecular Biosciences, College of Veterinary Medicine, Washington State University, Pullman, WA, United States

**Keywords:** *Chlamydia trachomatis*, fibrosis, EMT, ECM, host-pathogen interaction, pathogenesis

## Abstract

Ocular, genital, and anogenital infection by the obligate intracellular pathogen *Chlamydia trachomatis* have been consistently associated with scar-forming sequelae. In cases of chronic or repeated infection of the female genital tract, infection-associated fibrosis of the fallopian tubes can result in ectopic pregnancy or infertility. In light of this urgent concern to public health, the underlying mechanism of *C. trachomatis*-associated scarring is a topic of ongoing study. Fibrosis is understood to be an outcome of persistent injury and/or dysregulated wound healing, in which an aberrantly activated myofibroblast population mediates hypertrophic remodeling of the basement membrane *via* deposition of collagens and other components of the extracellular matrix, as well as induction of epithelial cell proliferation *via* growth factor signaling. Initial study of infection-associated immune cell recruitment and pro-inflammatory signaling have suggested the cellular paradigm of chlamydial pathogenesis, wherein inflammation-associated tissue damage and fibrosis are the indirect result of an immune response to the pathogen initiated by host epithelial cells. However, recent work has revealed more direct routes by which *C. trachomatis* may induce scarring, such as infection-associated induction of growth factor signaling and pro-fibrotic remodeling of the extracellular matrix. Additionally, *C. trachomatis* infection has been shown to induce an epithelial-to-mesenchymal transition in host epithelial cells, prompting transdifferentiation into a myofibroblast-like phenotype. In this review, we summarize the field’s current understanding of *Chlamydia*-associated fibrosis, reviewing key new findings and identifying opportunities for further research.

## 1 Introduction

The phylum *Chlamydiae* comprises a group of Gram-negative, obligate intracellular pathogens and symbiotes targeting a variety of host organisms, from single-celled amoebae to humans (Bachmann et al., 2014). The member family Chlamydiaceae includes a variety of pathogenic species distinguished by host preference, such as *Chlamydia muridarum* (mice)*, Chlamydia psittaci* (birds), *Chlamydia suis* (pigs), and the human-infecting species *Chlamydia pneumoniae* and *Chlamydia trachomatis*. Of these, *C. trachomatis* bears particular infamy as the most common bacterial sexually transmitted infection worldwide. While the *C. trachomatis* biovars exhibit stark differences in the epithelial mucosae they infect, it is critical to note that conjunctival, urogenital, and anogenital infections caused by this pathogen are universally associated with fibrotic pathology. Chronic ocular infection by *C. trachomatis* serovars A-C results in both conjunctival inflammation and extensive collagen deposition, resulting in trichiasis (inward turning of the eyelid), leading to blindness due to progressive abrasion of the cornea by the eyelashes (Solomon et al., 2022). In similar fashion, chronic genital infection by *C. trachomatis* serovars D-K can promote scarring-associated infertility. Infection of the upper female genital tract is consistently associated with chronic inflammation as well as fibrosis-related blockage of the fallopian tubes (Chow et al., 1990; Passos et al., 2022). Long-term infection by the lymphogranuloma venereum serovars L1-L3 produces similar fibrotic obstruction of the lymphatic system – in severe cases resulting in rectal strictures, fistulae, or elephantiasis of the genitalia (Mabey and Peeling, 2002; Ceovic and Gulin, 2015). Given the consistent incidence of fibrotic sequelae associated with *C. trachomatis* infection, a principal goal of the field has been the elucidation of the mechanisms underlying chlamydial fibrosis.

**Figure 1:**
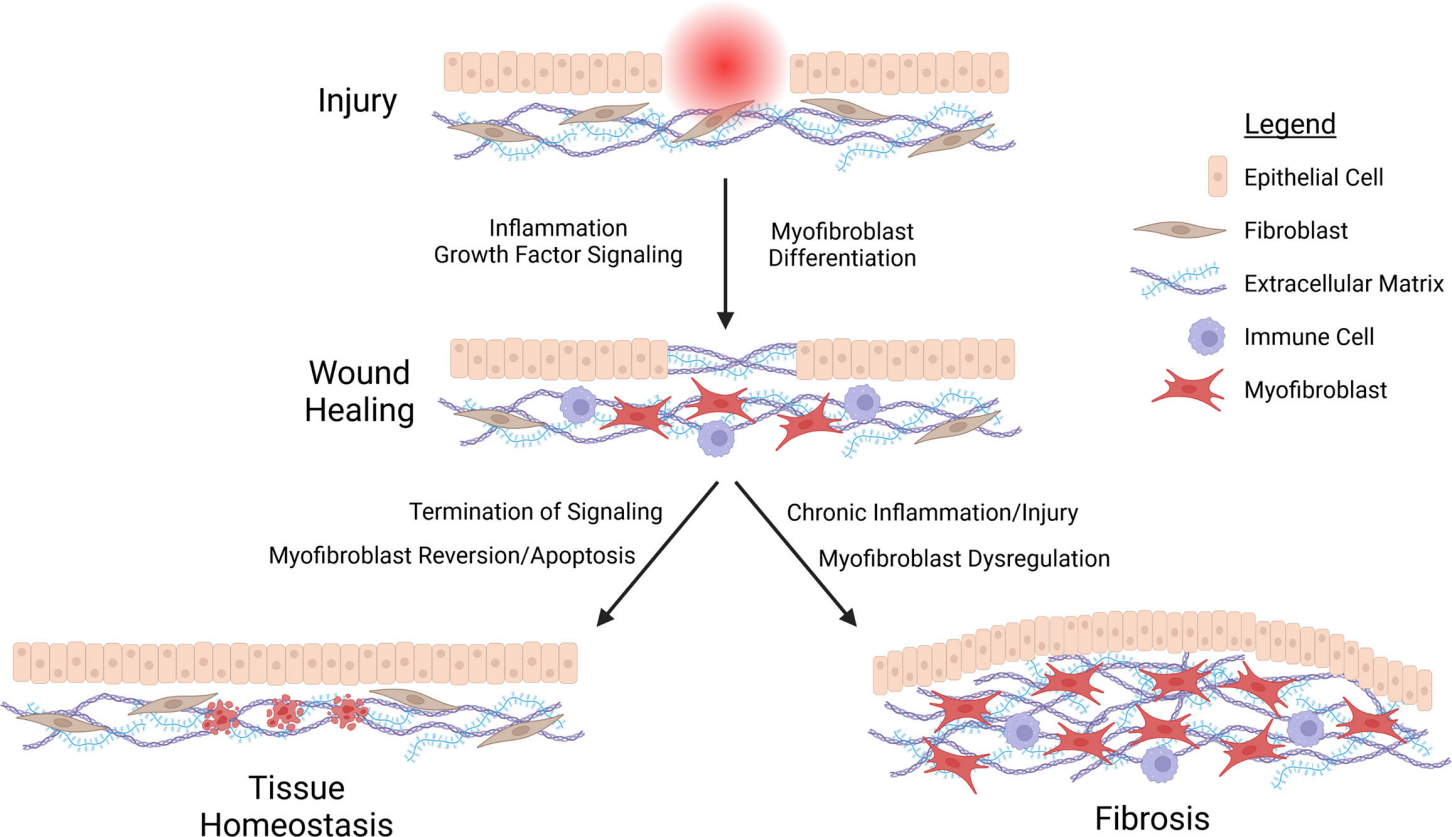
Schematic representation of fibrosis. Injury stimulates the recruitment of immune cells and differentiation of myofibroblasts at the wound side, facilitating the deposition of a provisional extracellular matrix (ECM) acting as a scaffold for epithelial cell migration and proliferation in closing the wound. Wound healing terminates with the cessation of inflammation and growth factor signaling, prompting reversion or apoptosis of myofibroblasts. Chronic inflammation or repeated injury leads to persistent myofibroblast activation. This in turn promotes excessive deposition of ECM components, epithelial cell proliferation, and ultimately fibrosis.

Fibrosis can be understood as an aberrant form of wound healing, wherein the physiological processes responsible for tissue repair are chronically activated by repeated injury and attendant inflammation, or by excessive induction of TGF-β and other mediators of growth factor signaling (Wynn, 2008; Wynn and Ramalingam, 2012; Distler et al., 2019; Henderson et al., 2020; Tsou et al., 2021). In the case of a typical injury by wounding, epithelial cells initiate a pro-inflammatory, fibrinogenic signaling cascade, prompting formation of a provisional scaffold of extracellular matrix (ECM) proteins that facilitates clotting (Wynn, 2008). Inflammation at the wound site promotes and is propagated by immune cell infiltration. Inflammation is dampened, which is critical for the initiation of the repair process; and repair is associated with the accumulation of myofibroblasts. This cell type mediates both wound healing and fibrosis. They are the principal cell type responsible for deposition of collagens, fibronectins, and other ECM and ECM-remodeling components (McAnulty, 2007). Myofibroblasts also mediate puckering of the wound site, through the purse-string type of contraction of cytoskeletal stress fibers rich in smooth muscle actin (α-SMA) (McAnulty, 2007). Myofibroblasts can arise from activation by TGF-β and other pro-fibrotic signaling in a variety of cell types, such as tissue-resident fibroblasts, pericytes, or smooth muscle cells (McAnulty, 2007; Distler et al., 2019; Tsou et al., 2021). Critically, epithelial cells can also be converted into myofibroblasts *via* a reversible form of trans-differentiation termed the epithelial-to-mesenchymal transition (EMT) (McAnulty, 2007; Dongre and Weinberg, 2019). Upon wound closure, myofibroblasts undergo programmed cell death or dedifferentiation, events that are coincident with termination of proliferative/fibrinogenic signaling (Distler et al., 2019; Tsou et al., 2021). However, in cases of repeated injury, chronic inflammation, or aberrant induction of growth factor signaling, myofibroblasts persist, leading to excessive deposition of collagens, epithelial cell proliferation due to overproduction of growth factors, and the consequent formation of a scar (**Figure 1**).

Initial work characterizing the extensive pro-inflammatory host response to *Chlamydia* in both animal and cell culture models of infection suggested that chlamydial fibrosis was the product of immune-mediated tissue damage (Grayston et al., 1985; Brunham and Peeling, 1994). Given that fibrosis is generally understood to arise from dysregulation of cell- and tissue-level processes regulating wound healing, the investigative focus on the inflammatory response to *Chlamydia* as a fibrosis-inciting event is understandable. Until recently, the prevailing hypothesis in the field posited that infection-associated sequelae are the product of this immune response, initiated by *Chlamydia*-infected epithelial cells (Stephens, 2003). Termed the cellular paradigm of chlamydial pathogenesis, this model effectively describes the role of epithelial cells in the initiation of inflammation, and associated subsequent scarring. However, the cellular paradigm falls short of explaining the high incidence of unreported and asymptomatic *C. trachomatis* infections resulting in fibrotic sequelae – where the inflammatory response to infection is either absent, or attenuated to the extent that it does not present clinically.

How does scar-forming disease arise in subclinical *C. trachomatis* infection? One potential explanation supported by the data is that infection itself stimulates scar formation. Early study of the proinflammatory response to *Chlamydia* also indicated that infection also induces production of fibrosis-associated cytokines such as IL-6, IL-11, and IL-17 (Rasmussen et al., 1997; Dessus-Babus et al., 2002; Jha et al., 2011; Masson et al., 2015; Johnson et al., 2020), as well as an extensive portfolio of fibrosis-associated growth factors (e.g. VEGF, CTGF, EGF) (Hess et al., 2001; Gonzalez-Moreno et al., 2010; Lipson et al., 2012; Zhu et al., 2015; Barratt et al., 2018; Adami et al., 2021; Widjaja et al., 2021). It has been additionally observed that infection can induce EMT in host epithelial cells (Igietseme et al., 2015; Rajić et al., 2017; Igietseme et al., 2018). Recent reports indicate that multiple *C. trachomatis* biovars stimulate expression of ECM components and maintenance enzymes in host epithelial cells (Humphrys et al., 2013; Porcella et al., 2015), as well as infection-associated EMT inducing production of collagen I (Igietseme et al., 2018). Collectively, these data imply an extension to the cellular paradigm of chlamydial pathogenesis, wherein *Chlamydia*-infected epithelial cells initiate pro-inflammatory and pro-fibrotic signaling simultaneously. In this review, we summarize the field’s current understanding of *Chlamydia*-associated fibrosis, identifying knowledge gaps and unanswered questions for future investigation.

## 2 *C. trachomatis* infection induces inflammation and immune cell infiltration

The conventional model of inflammation-mediated chlamydial fibrosis arose from an early observation that tissues infected with *C. trachomatis* and related species exhibit the swelling and mucosal discharge associated with an inflammatory response (Stephens, 2003; Barron, 2019). Indeed, *C. trachomatis* infection of both the ocular and genital tract is associated with robust infiltration of affected tissues by the innate and adaptive immune systems (Barron, 2019). The initial immune response to *Chlamydia* is characterized by recruitment of polymorphonuclear neutrophils and lymphocytes, followed by infiltration of plasma cells and macrophages (Barron, 2019). Subsequent development of lymphoid follicles containing B-cells, T-cells, and macrophages presumably facilitates antigen processing and presentation facilitating the induction of an adaptive immune response to infection (Taylor et al., 1982; Patton and Taylor, 1986). Critically, animal models of chlamydial infection employing *C. pneumoniae* exhibit a similar dynamic of neutrophil and macrophage recruitment at infection sites, suggesting the host response to *Chlamydia* is conserved between species (Moazed et al., 1996; Fong et al., 1997; Fong et al., 1999). Combined with epidemiological and animal model data demonstrating the increased risk for fibrotic sequelae conferred by repeated infections (Grayston et al., 1985), these early observations suggested an initial, immunological mechanism of chlamydial pathology, wherein chlamydial antigen recognition by the adaptive immune system promotes inflammation *via* either delayed hypersensitivity or autoimmunity. In this proposed model of *Chlamydia*-associated fibrosis, an overactive adaptive immune response to the pathogen leads indiscriminate tissue damage, to which the host reacts by initiating tissue repair.

A molecular basis for fibrosis driven by chlamydial antigen recognition has proved elusive. An early candidate for an antigen driving the inflammatory response was the chlamydial homolog of Hsp60, which was implicated in the induction of a delayed-type hypersensitivity reaction *via* treatment of *Chlamydia-*immunized guinea pigs with chlamydial protein extracts prepared with the detergent Triton X-100 (Watkins et al., 1986; Arno et al., 1995). Given that serum reactivity to chlamydial Hsp60 was shown to correlate with infection-associated sequelae (Arno et al., 1995; Eckert et al., 1997; Peeling et al., 1997), it was postulated that *C. trachomatis* infection may induce autoimmunity *via* molecular mimicry. However, the unfortunate discovery that administration of Triton X-100 alone was sufficient to induce inflammation brought Hsp60’s relevance into question (Taylor et al., 1987). Indeed, more detailed study of chlamydial Hsp60 reactivity in convalescent sera did not reveal a shared epitope mimicking human Hsp60 (Yi et al., 1997; Sziller et al., 1998), suggesting that the presence of Hsp60-reactive antibodies is only a biomarker of chronic infection, not an indicator of autoimmunity. To complicate matters further, reports positively correlating trachoma with a humoral, Th2-mediated immune response seemingly dismissed delayed hypersensitivity to as a potential mechanism of chlamydial fibrosis as well (Holland et al., 1993; Bailey et al., 1995; Holland et al., 1996).

While these data suggest immune recognition of chlamydial Hsp60 does not lead to the development of autoimmunity or delayed hypersensitivity, recent work indicates this effector may still facilitate *Chlamydia*-associated fibrosis indirectly, as an inducer of inflammation (Raulston et al., 1998; Bulut et al., 2002; Costa et al., 2002). Importantly, antibodies against chlamydial Hsp60 are consistently associated with chronic infection and *Chlamydia*-associated infertility (Hjelholt et al., 2011). Given the demonstrable importance of CD4^+^ T-cells to clearance of the pathogen (Su and Caldwell, 1995; Gondek et al., 2012)*¸* these data suggest that antigenic recognition of chlamydial Hsp60 enhances the inflammatory response to infection, even if Hsp60-targeting T-cells are not autoreactive. Additionally, Hsp60 has also been shown to induce an innate immune response in macrophages in a TLR4/Myd88-dependent fashion (Bulut et al., 2002), illustrating a complementary mechanism by which this chlamydial protein may act as a proinflammatory stimulus independent of inducing autoimmunity or hypersensitivity. Similarly, Hep2 cells infected with *C. pneumoniae* have been observed to activate murine dendritic cells in a TLR2/4-dependent fashion analogous to treatment with recombinant chlamydial Hsp60 (Costa et al., 2002). Importantly, Hsp60 is retained in an *in vitro* model of infection for up to 14 days after clearance of the pathogen (Raulston et al., 1998), implying that infection-associated inflammation *via* recognition of Hsp60 may persist after infection as well. Given these data, it is tempting to speculate that Hsp60 acts as a chronic pro-inflammatory stimulus in infected tissues, thereby facilitating the development of fibrosis. That said, it remains unclear to what degree Hsp60 expression by *Chlamydia* is required for induction of infection-associated inflammation. Leveraging recent advances in chlamydial genetics for conditional knockout or knockdown of Hsp60 is thus likely to reveal the importance of this protein to induction of the inflammatory response to *Chlamydia* – and, by extension, its relevance to the development of *Chlamydia*-associated fibrosis).

## 3 Fibrotic sequelae as a consequence of the host cell response to infection

Stephens’s seminal 2003 review articulated an alternative hypothesis of chlamydial pathogenesis, centering infected epithelial cells as principal initiators of the host response to *Chlamydia* (Stephens, 2003). A model of infection wherein non-immune, *Chlamydia*-infected epithelial cells recruit immune cells into affected tissues is uniquely persuasive, given epithelial cells secrete a host of pro-inflammatory cytokines (e.g. IL-1α, IL-8, GROα, GM-CSF) in both murine and *in vitro* models of infection (Eckert et al., 1997; Rasmussen et al., 1997; Darville et al., 2001; Finethy et al., 2016). Infection of endothelial cells with *C. pneumoniae* similarly induces production of IL-8, also recruiting neutrophils and macrophages – reinforcing a model of host cell-mediated infection response that is conserved between species (Molestina et al., 1999). IL-17 has been shown to drive a Th1-mediated response to infection in similar fashion, with *C. muridarum*-infected *il17ra* ^-/-^ and *il17* ^-/-^ mice exhibiting diminished recruitment of neutrophils and/or macrophages (Scurlock et al., 2011; Andrew et al., 2013), likely due to the attenuated activity of known IL-17-producing cell types at the infection site (e.g. Th17, γδ+ T, NK cells) (Gaffen et al., 2014; Monin and Gaffen, 2018). A cellular paradigm of chlamydial pathogenesis could also explain epidemiological data linking repeat infections to increased risk of fibrotic sequelae (Davies et al., 2016), despite no clear consensus in reactivity to specific chlamydial antigens. Repeated infection would necessarily result in host cell recruitment of memory B/T-cells, no doubt exacerbating the inflammatory response. Indeed, complementary mechanisms by which *C. trachomatis* may induce fibrosis by providing pro-inflammatory stimuli have since been described (Raulston et al., 1998; Bulut et al., 2002; Stephens, 2003; Panzetta et al., 2018).

One such means by which *C. trachomatis* infection may facilitate a protracted immune response is *via* induction of a developmental state known as chlamydial persistence. Persistence is characterized by an aberrant, enlarged bacterial phenotype distinct from both EBs and RBs, associated with diminished bacterial replication and attendant reduction of infection-competent progeny (Panzetta et al., 2018). Evidence for persistence as a stress response was apparent as early as 1950, with reports of *C. muridarum* and *C. felis* (then called the murine/feline pneumonitis viruses) exhibiting enlarged morphologies in response to penicillin treatment (Weiss, 1950). Subsequent work showed a similar phenotype in LGV serovar *C. trachomatis* and *C. psittaci* infections *in vitro* (known then as lymphogranuloma and meningopneumonitis viruses, respectively), suggesting the presence of a conserved stress response amongst *Chlamydia* (Hurst et al., 1953; Matsumoto and Manire, 1970). In the case of *C. psittaci* infection, penicillin treatment attenuated production of infection-competent bacteria to as low as 0.1% of the original inoculum. Strikingly, bacterial replication recovered rapidly after penicillin was removed, even after more than three months of continuous treatment (Galasso and Manire, 1961). In this way, persistence can be understood as a survival mechanism wherein *Chlamydiae* encountering suboptimal (but not bacteriocidal) growth conditions halt replication and EB differentiation, until such time as growth conditions improve.

While the specific mechanisms underlying persistence are incompletely characterized, induction of persistence has been shown to occur in response to a variety of physiologically relevant stimuli. Critically, these include *in vitro* treatment of infected cells with the pro-inflammatory cytokine IFN-γ, which has the effect of inducing catabolism of tryptophan – an amino acid for which *C. trachomatis* is auxotrophic (Beatty et al., 1993; Beatty et al., 1994). As with penicillin, abrogation of IFN-γ treatment or supplementation with tryptophan prompts swift recovery of the pathogen’s replicative and infective capacity (Batteiger, 2012). Importantly, IFN-γ production is induced in *C. trachomatis* infection of the cervix and fallopian tubes (Reddy et al., 2004), suggesting that persistence may be relevant to infection of the female upper genital tract, and thereby provide an ongoing inflammatory stimulus potentially driving infection-associated fibrosis.

While it is tempting to attribute fibrotic sequelae in subclinical/asymptomatic infections to chlamydial persistence, the incidence of persistent *Chlamydia* in human infection is poorly understood, as is the pathogen’s capacity to recover from persistence-inducing stressors *in vivo*. Further, it remains unclear to what degree persistence states induced by varying stressors are comparable, and thus whether persistent *Chlamydia* arising from varying stressors possess an equivalent capacity to stimulate inflammation. Importantly, it is posited that iron and tryptophan limitation alter the chlamydial transcriptome *via* different mechanisms, with iron limitation inducing persistence *via* the action of iron-regulated transcription factors and iron-dependent enzymes (Raulston, 1997; Al-Younes et al., 2001), and tryptophan starvation altering the chlamydial proteome *via* the arrested translation of tryptophan-rich proteins (Ouellette et al., 2016). Accordingly, our laboratory recently observed that induction of persistence *via* iron and tryptophan starvation induced transcriptional responses in *Chlamydia* exhibiting incomplete overlap, comprised of a shared, “core” stress response as well as distinct “accessory” differential expression unique to each stressor (Pokorzynski et al., 2022). Taken together, these data illustrate a need to further characterize the chlamydial transcriptomic response to other known inducers of persistence, such as penicillin and IFN-γ – as well as to assess the degree to which persistent *Chlamydia* arising from varying stressors induce pro-inflammatory and pro-fibrotic responses in the host.

The cellular paradigm of chlamydial pathogenesis elegantly accounts for much of the work describing the host response to the pathogen, and the notion of persistence accounts for the chronic nature of infection, i.e. through reactivation to sustain the pro-inflammatory response by epithelial cells. Indeed, this model may account for the role of host epithelial cells in *Chlamydia*-associated fibrosis. However, key points of contradictory data prevent this hypothesis from completely explaining how *Chlamydia*-associated fibrosis occurs. First, the observation that fibrotic sequelae in conjunctival infections (i.e. trichiasis) correlate with a predominantly humoral, Th2-mediated response to infection is seemingly in conflict with the assertion that initiation of a cell-mediated response by infected tissues underlies fibrotic pathology (Holland et al., 1993; Bailey et al., 1995; Holland et al., 1996). Accordingly, it has been observed that a Th2-mediated response to genital infection in mice exhibits ineffective clearance of *C. muridarum*, with mice receiving anti-*Chlamydia* Th2 cells exhibiting increased bacterial titer relative to those receiving anti-*Chlamydia* Th1 cells despite demonstrable production of anti-*Chlamydia* antibodies (Hawkins et al., 2002). Combined with the observation that *C*. *muridarum*-infected infertile mice exhibit increased induction of Th2-associated cytokines relative to fertile mice (Igietseme et al., 2017), these data call suggest that cell-mediated immunity may not be the sole driver of *Chlamydia*-associated fibrotic sequelae *in vivo*. Given estimates that up to 75% of *C. trachomatis* infections go unreported due to absent or subclinical symptoms – and that up to 18% of such cases are believed to cause infertility – any comprehensive model of pathology must also account for infections where attenuated or absent inflammation nevertheless results in fibrosis (Haggerty et al., 2010). Indeed, Stephens acknowledges this possibility in noting that *Chlamydia*-infected epithelial cells produce the pro-fibrotic cytokines IL-6 and IL-11 (Rasmussen et al., 1997; Dessus-Babus et al., 2000; Dessus-Babus et al., 2002), and induce expression of growth factors like VEGF, EGF, and CTGF (Coombes and Mahony, 2001; Hess et al., 2001). Though not necessarily produced by host epithelial cells, genital infection by *C. trachomatis* is also associated with production of IL-17 (Jha et al., 2011; Masson et al., 2015), which has been shown to enhance or inhibit other forms of fibrotic disease (Ramani and Biswas, 2019). Collectively, these results propose an expansion to the cellular paradigm of chlamydial pathogenesis, wherein infected epithelial cells initiate both pro-inflammatory signaling and pro-fibrotic tissue remodeling.

## 4 Chlamydial induction of the epithelial-to-mesenchymal transition

Supporting this hypothesis are recent reports of *Chlamydia*-infected epithelial cells exhibiting pro-fibrotic phenotypes (**Table 1**), including an apparent epithelial-to-mesenchymal transition (EMT). EMT is a reversible process of trans-differentiation, where epithelial cells progressively transform their morphological and functional properties into those of mesenchymal fibroblasts (Dongre and Weinberg, 2019). Typically, EMT involves the loss of cell-cell adhesions due to transcriptional repression of epithelial cadherin (E-Cadherin), prompting the disassembly of adherens junctions (Kalluri and Weinberg, 2009). This in turn leads into loss of the apical-basal polarity, cobblestone morphology, and cytokeratin expression associated with epithelial differentiation, in favor of a spindle-shaped phenotype characterized by front-rear polarity, reorganization of the extracellular matrix (ECM), and expression of mesenchymal markers (Lamouille et al., 2014; Shibue and Weinberg, 2017; Dongre and Weinberg, 2019). EMT-associated mesenchymal markers include neural cadherin (N-cadherin), the intermediate filament vimentin, extracellular matrix (ECM) components and maintenance enzymes (e.g. fibronectin, matrix metallopeptidases, collagens), as well as the alpha isoform of smooth muscle actin (α-SMA) (Kalluri and Weinberg, 2009; Thiery et al., 2009). Induction of EMT has been reported in a variety of physiological contexts, leading to three context-specific subclassifications: type 1 EMT is associated with embryogenesis and organ/tissue development, type 2 EMT occurs in the context of wound healing and scar formation, and type 3 EMT is implicated in the metastatic progression of cancer (Kalluri and Weinberg, 2009).

**Table 1:** Fibrosis and EMT-associated molecules induced in host epithelial cells by *C. trachomatis* infection.

Name	Function	Role(s) in Fibrosis	References
IL-6	Cytokine	Myofibroblast differentiation, collagen deposition, EMT induction	Rasmussen et al., 1997
IL-8	Cytokine	Mesenchymal proliferation, EMT induction	Rasmussen et al., 1997
IL-11	Cytokine	TGFβ signal transduction, collagen deposition	Dessus-Babus et al., 2002
VEGF	Growth Factor	Collagen deposition, EMT induction	Hess et al., 2001
CTGF	Growth Factor	Myofibroblast differentiation, collagen deposition, EMT induction	Coombes and Mahony, 2001
EGF	Growth Factor	Epithelial cell proliferation, induction of EMT	Coombes and Mahony, 2001
miR-9	microRNA	Induction of EMT	Igietseme et al., 2015
miR-15a	microRNA	Repression of EMT	Igietseme et al., 2015
miR-16	microRNA	Repression of EMT	Igietseme et al., 2015
miR-22	microRNA	Induction of EMT	Igietseme et al., 2015
ZEB1	Transcription Factor	Master regulator of EMT induction, E-cadherin repression	Igietseme et al., 2015
ERF	Transcription Factor	EMT induction	Zadora et al., 2019
ETS1	Transcription Factor	Induction of EMT	Zadora et al., 2019
YAP	Transcription Factor	Induction of EMT, CTGF expression, TGFβ signaling	Caven et al., 2023
FRA1	Transcription Factor	Induction of EMT	Zadora et al., 2019
MAPK	Kinase	Stabilization of SNAIL, TGFβ-mediated EMT induction	Zadora et al., 2019
ERK	Kinase	Stabilization of SNAIL, TGFβ-mediated EMT induction	Zadora et al., 2019
α-SMA	Actin Cytoskeleton	Fibroblast contractility, marker of myofibroblast differentiation	Rajić et al., 2017
OLFM4	ECM	Repression of EMT	Kessler et al., 2012
Fibronectin	ECM	ECM deposition, marker of mesenchymal differentiation	Igietseme et al., 2018
Collagen I	ECM	ECM deposition, induction of myofibroblast activation	Igietseme et al., 2018
Collagen III	ECM	ECM deposition, induction of myofibroblast activation	Igietseme et al., 2018
MMP2	ECM	Collagen degradation, matrikine production	Ault et al., 2002
ADAM12	ECM	Enzymatic cleavage of type IV collagen, fibronectin, gelatin	Humphrys et al., 2013
ADAM19	ECM	Collagen synthesis	Humphrys et al., 2013
ADAMTS3	ECM	Enzymatic cleavage of procollagen II	Humphrys et al., 2013

EMT can be initiated by a variety of physiological processes, including both SMAD-dependent and independent signaling of TGFβ-family growth factors (Portella et al., 1998; Valcourt et al., 2005; Lamouille and Derynck, 2007; Lamouille et al., 2014), the Wnt/β-catenin signaling pathway (Basu et al., 2018), growth factor signaling *via* receptor tyrosine kinases (e.g. EGF, PDGF) (Lu et al., 2003; Yang et al., 2006; Lo et al., 2007), and regulation of gene expression by non-coding miRNAs (Gregory et al., 2008; Ma et al., 2010; Ru et al., 2012). However, common to all of these modes of induction is the activity of EMT-associated “master regulators” that drive transcriptional repression of genes associated with epithelial differentiation, and/or induction of genes associated with mesenchymal differentiation. Chief among these are the SNAIL and zinc-finger E-box-binding (ZEB) families of transcription factors (Sánchez-Tilló et al., 2010; Lamouille et al., 2014). SNAIL1 (also known as SNAI1, or simply SNAIL) has been shown to directly bind to the promoter region of E-cadherin, recruiting the PRC2 complex of histone-modifying enzymes and subsequently facilitating heterochromatin formation and transcriptional silencing (Batlle et al., 2000; Peinado et al., 2004; Herranz et al., 2008; Lin et al., 2010, 1). SNAIL2 (also known as SNAI2 or SLUG) binds to the proximal E-box of the E-cadherin promoter region as well, acting to inhibit E-cadherin expression through a presumably similar mechanism (Bolós et al., 2003). ZEB1 has been shown to also inhibit E-cadherin expression in a similar fashion, either *via* recruitment of a C-terminal binding protein (CTBP) co-repressor, or SWI/SNF remodeling of chromatin mediated by BRG1 (Sánchez-Tilló et al., 2010, 1; Lamouille et al., 2014). Critically, ZEB1 expression in EMT typically occurs subsequent to that of SNAIL. Given data indicating SNAIL binds to the promoter regions of ZEB1/ZEB2 and can regulate their expression, this suggests a model centering on the induction of SNAIL as a key upstream event in the initiation of EMT (Peinado et al., 2007; Dave et al., 2011; Lamouille et al., 2014).

Evidence suggesting that *C. trachomatis* could mediate an EMT-like phenotype was initially apparent in *ex vivo* infection of fallopian tube organ cultures, with the observation that infection impaired cell-cell adhesion *via* sequestration of β-catenin to the inclusion surface and subsequent induction of Wnt-mediated paracrine signaling (Kessler et al., 2012). While the authors did not associate this phenotype with EMT – indeed, reporting the infection-associated induction of known EMT antagonists such as OLFM4 (Li et al., 2019) – these data nevertheless suggest the potential for infection to disrupt epithelial homeostasis through the induction of known EMT-inducing pathways. Chlamydia-associated EMT was first demonstrated outright in Igietseme et al.’s landmark 2015 study, which reported that the oviducts of mice infected intravaginally with *C. trachomatis* serovar L2 exhibited differential expression of EMT-associated miRNAs (Igietseme et al., 2015). This included both downregulation of known EMT-repressing miRNAs (e.g. miR-15a, miR-16) as well as upregulation of known EMT-inducing miRNAs (e.g. miR-9, miR-22) (Ma et al., 2010; Song et al., 2013; Igietseme et al., 2015; Renjie and Haiqian, 2015). The authors further characterized the chlamydial EMT phenotype *via* an *in vitro* model of infection using primary epithelial cells of the murine oviduct, revealing infection-dependent repression of E-cadherin and induction of SNAIL1/2, fibronectin, and ZEB1 *via* immunofluorescence. Importantly, this phenotype was sensitive to pan-caspase inhibition *via* treatment with Z-VAD-fmk, reinforcing the authors’ hypothesis that caspase-dependent inhibition of the RNA-cleaving enzyme dicer and attendant dysregulation of miRNA was responsible for chlamydial EMT (Igietseme et al., 2015). A subsequent report from this laboratory confirmed that the chlamydial EMT-like phenotype resulted in myofibroblast differentiation: murine oviduct epithelial cells infected with *C. trachomatis* serovar D exhibited striking expression of α-SMA, as well as repression of the epithelial differentiation marker β-catenin (Igietseme et al., 2018).

Further work has implied that infection induces EMT *via* multiple, complementary processes. For example, a 2017 report suggested ocular *C. trachomatis* infection may induce EMT *via* altering host DNA methylation, correlating diminished E-cadherin and increased fibronectin/α-SMA with an altered methylome in serovar B-infected human conjunctival epithelial cells, including increased CpG methylation of the E-cadherin promoter (Rajić et al., 2017). Zadora et al. demonstrated that *C. trachomatis* infection of End1/E6E7 immortalized endocervical epithelial cells extensively altered the host phosphoproteome, including the enhancement of mitogen-activated protein kinase (MAPK), extracellular signal-related kinases (ERK), and pro-EMT transcription factors ERF, ETS1, and FRA1 (Allegra et al., 2012; Bakiri et al., 2015; Li et al., 2015; Zadora et al., 2019). MAPK/ERK activity is critical to multiple stages of EMT progression, including stabilization of SNAIL, SMAD-independent induction of EMT by TGF-β, as well as downstream EMT activation by EGF engagement with receptor tyrosine kinases (Lamouille et al., 2014). Accordingly, infection-dependent phosphoactivation of ERF/ETS1/FRA1 was sensitive to attenuation of ERK/MAPK activity *via* treatment with the small-molecule inhibitor U0126 (Zadora et al., 2019). This study further confirmed the initial EMT-like phenotype reported by Igietseme et al., demonstrating E-cadherin repression, attendant loss of cell-cell adhesion, and increased cell motility/invasiveness after 7 days of persistent *C. trachomatis* infection in three-dimensional tissue culture (Zadora et al., 2019). The invasive properties of infected cells were significantly inhibited by expression of ERF loss-of-function mutants or CRISPR-mediated knockout of ETS1, reinforcing the authors’ hypothesis that ERK-mediated enhancement of these transcription factors contributes to chlamydial induction of EMT (Zadora et al., 2019). Subsequent work from the Igietseme laboratory has shown that *C. muridarum* infection can rapidly induce expression of TGF-β *via* EB engagement with the epidermal growth factor receptor (EGFR) during invasion (Igietseme et al., 2020). Pharmacological inhibition of either the TGF-β or EGF receptors (using SB-431542 or Gefitinib/ZD-1839, respectively) significantly attenuated infection-dependent repression of E-cadherin, suggesting that *Chlamydia*-induced TGF-β may act in an autocrine fashion on infected cells to promote EMT (Igietseme et al., 2020). Collectively, these reports indicate that *Chlamydia* induces an epithelial-to-mesenchymal transition in the host *via* multiple, functionally redundant methods of co-opted signal transduction – with significant implications for the development of scarring independent of immune cell recruitment.

## 5 Infection*-*dependent modulation of the extracellular matrix

Increased deposition of extracellular matrix (ECM) components is a critical hallmark of fibrotic tissues, which consistently exhibit hypertrophic basement membranes with excessive accumulation of component proteins (Wynn, 2008; Karsdal et al., 2017). In healthy epithelial tissues, the ECM of the basement membrane is generally composed of network-forming type IV collagen, laminin, and the collagen/laminin crosslinker nidogen (Schuppan, 1990; Van Agtmael and Bruckner-Tuderman, 2009). At sites of tissue injury, pro-inflammatory and growth signaling stimulates myofibroblast differentiation and subsequent production of fibrillar collagens (e.g. collagen I, II, III, V). Dysregulated activation of myofibroblasts has been similarly associated with fibrillar collagen deposition; indeed, an early morphological study of idiopathic pulmonary fibrosis using a monoclonal collagen I antibody revealed a core of nonproliferating myofibroblasts as the principal locus of ECM synthesis (Kuhn and McDonald, 1991). Excessive collagen III deposition has been similarly associated with the progression of IPF and other forms of fibrotic disease (Kuhn et al., 1989; Karsdal et al., 2017). The proportionality of specific collagens in the ECM is maintained by multiple, complementary modes of regulation, including modulation of collagen gene expression, trafficking of procollagens by intracellular chaperones (e.g. Hsp47/90), processing of procollagens into mature collagen (e.g. *via* cleavage by ADAMTS-family proteins or BMP1), and proteolytic turnover of mature collagen by matrix metalloproteinases/metallopeptidases (MMPs) (Wynn, 2008; Wight and Potter-Perigo, 2011; Karsdal et al., 2017). Mounting evidence indicates that the ECM serves more than a structural function: the composition and architecture of the ECM can itself induce further pro-fibrotic activation of epithelial cells and tissue-resident fibroblasts, thereby driving progression of scarring (Wight and Potter-Perigo, 2011; Karsdal et al., 2017; Herrera et al., 2018).

**Figure 2:**
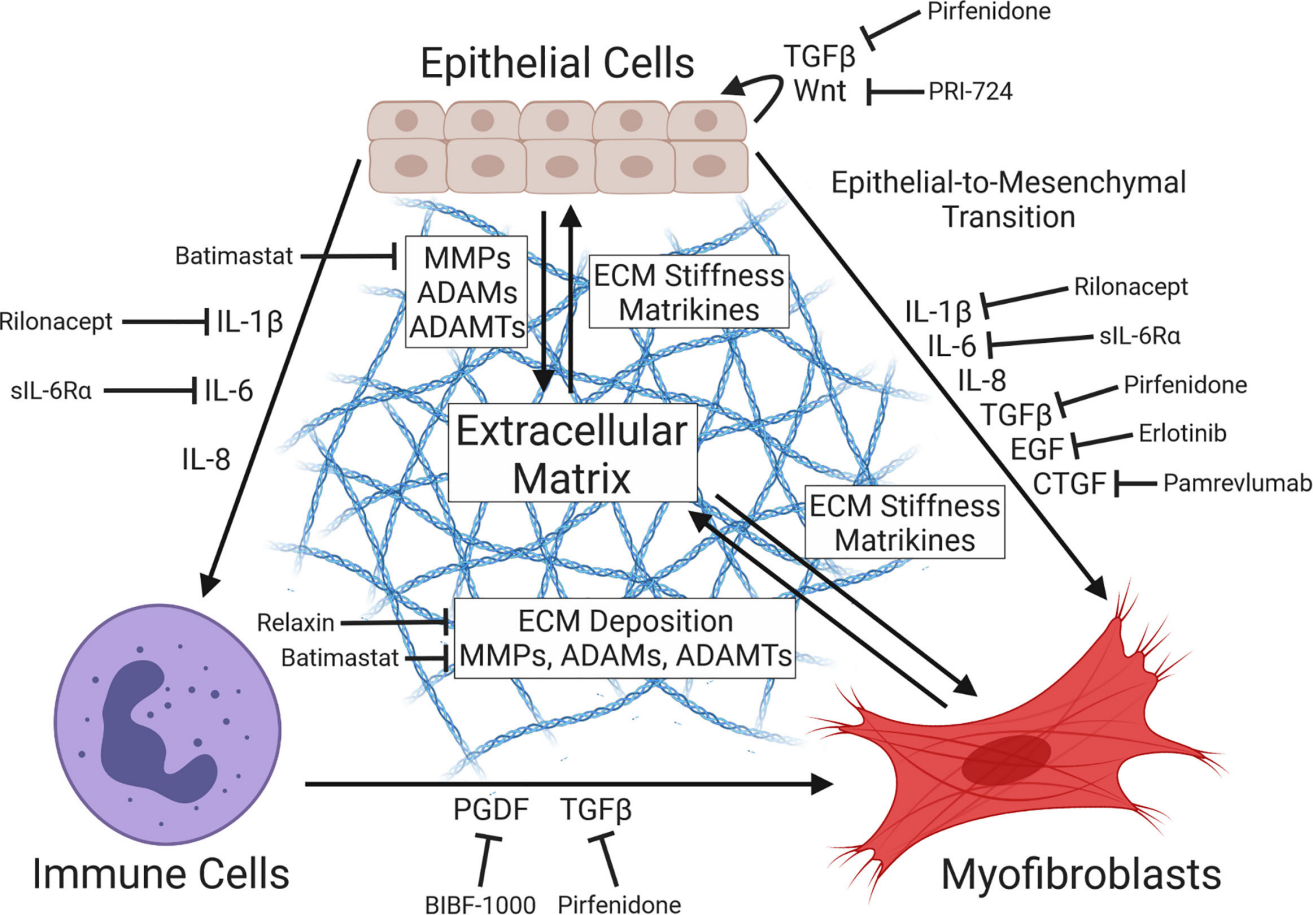
Diagram of pro-fibrotic intercellular communication. Tissue injury prompts secretion of signal factors at the wound site, including pro-inflammatory cytokines (e.g. IL-1β, IL-6/8) and growth factors (e.g. TGFβ, EGF, CTGF), driving the proliferation of activated myofibroblasts *via* induction of fibroblast differentiation or epithelial-to-mesenchymal transition (EMT). Cytokine-mediated recruitment of immune cells (e.g. M.2 macrophages, neutrophils, dendritic cells) and their subsequent expression of myofibroblast-activating signal factors contributes to this effect. Deposition of collagens and other extracellular matrix (ECM) components by myofibroblasts and ECM-restructuring enzymes by both epithelial cells and myofibroblasts produces basement membrane stiffening and pro-fibrotic cytokines, further driving myofibroblast activation in a positive feedback loop *via* the action of ECM-derived matrikines and mechanotransduction. Importantly, multiple inhibitors of fibrosis-associated signaling have been identified (Li et al., 2017; Zhao et al., 2022), including the TGFβ antagonist pirfenidone (Antoniu, 2006), the EGFR inhibitor erlotinib (Fuchs et al., 2014), the anti-CTGF recombinant antibody pamrevlumab (Richeldi et al., 2020), the PGDF receptor kinase inhibitor BIBF-1000 (Chaudhary et al., 2007), the myofibroblast-inhibitory hormone relaxin (Samuel et al., 2014; Huuskes et al., 2015), the Wnt/β-catenin antagonist PRI-724 (Akcora et al., 2018), the MMP inhibitor batimastat (Corbel et al., 2001), the IL-1βR antagonist rilonacept (Li et al., 2017), and the soluble IL-6 receptor sIL-6Rα (Le et al., 2014).

Once liberated from the ECM by proteolysis, fragments of collagen, fibrin, fibronectin, and hyaluronan have been shown to act as signaling ligands known as matrikines (Genovese and Karsdal, 2016; Karsdal et al., 2017). For example, *N-*acetyl proline-glycine-proline (ac-PGP) produced by MMP-mediated destruction of collagens has been shown to act as a leukocyte chemoattractant, as well as inducing expression of MMP-9 and inflammation *via* binding to the CXC-chemokine receptor complex CXCR1/2 (Rodríguez et al., 2010; Akthar et al., 2015). In similar fashion, proteolytic cleavage of collagen I by MMP-2/9 can generate matricryptin, a pro-angiogenic signaling factor implicated in the progression of cardiac fibrosis (Lindsey et al., 2015). Fibrosis-associated ECM remodeling has the additional effect of increasing the stiffness of the basement membrane, which is similarly implicated in pro-fibrotic signaling. The process by which substrate stiffness, cytoskeletal tension, and other mechanical cues influence gene expression – termed mechanotransduction – has been shown to modulate cell proliferation and differentiation (Herrera et al., 2018; Tschumperlin et al., 2018). One mediator of this effect is the transcriptional cofactor YAP (Yes-associated protein), whose access to the nuclear compartment (and, by extension, ability to modulate gene expression) is demonstrably regulated by binding of cell surface integrins to ECM components, cell substrate stiffness, and cell-cell contact (Finch-Edmondson and Sudol, 2016). Importantly, we recently observed the pathogen-directed induction of YAP *via* increased nuclear translocation during *C. trachomatis* infection of endocervical epithelial cells, suggesting that infection may facilitate fibrosis through manipulation of this transcription factor (Caven et al., 2023). Importantly, dysregulation of YAP has been associated with increased TGF-β signaling, myofibroblast differentiation, and induction of EMT (Liu et al., 2015; Szeto et al., 2016; Park et al., 2019). The myocardin-related transcription factors A/B (MRTF-A/B) function similarly – sequestered in the cytoplasm upon binding to monomeric actin, MRTF-A/B are induced in response to cytoskeletal remodeling and increased substrate stiffness, driving induction of similar pro-fibrotic outcomes (Huang et al., 2012; Scharenberg et al., 2014; Gasparics and Sebe, 2018). These data collectively illustrate how remodeling of the ECM can potentially exacerbate pro-fibrotic signaling *via* the creation of a positive feedback loop, mediated by matrikines liberated *via* ECM proteolysis, as well as mechanotransduction associated with increased stiffness of the basement membrane (**Figure 2**). Importantly, ECM-mediated induction of fibrosis is likely to occur independent of the immune response to *Chlamydia*, thus constituting a potential means by which subclinical or asymptomatic infections promote the development of fibrotic sequelae.

While it is unclear to what extent *Chlamydia*-associated scarring acts *via* ECM-mediated signaling, preliminary evidence indicates that *Chlamydia*-infected epithelial cells may alter ECM architecture. In their study further characterizing chlamydial induction of EMT, Igietseme et al. demonstrated that myofibroblast differentiation of *C. trachomatis*-infected murine oviduct epithelial cells at 48 hpi was additionally associated with production of pro-fibrotic ECM components, such as type I/III collagen and fibronectin (Igietseme et al., 2018). Critically, collagen expression was sensitive to caspase inhibition *via* Z-VAD-fmk treatment (Igietseme et al., 2018), implying that infection-associated ECM deposition was driven by the same mechanism of caspase-associated inhibition of dicer (and consequent dysregulation of host miRNAs) put forward in the authors’ original study (Igietseme et al., 2015). In an *in vitro* model of infection using human fallopian tube explants, *C. trachomatis* infection induced production of MMP2 and MMP9 by infected epithelial and stromal cell populations, respectively (Ault et al., 2002). The clinical relevance of stromal MMP9 production is unclear, given the epithelial tissue tropism of *C. trachomatis* infections *in vivo*; however, MMP2 secretion by infected epithelial cells may facilitate scar formation *via* proteolytic remodeling of the ECM and the production of pro-fibrotic matrikines. Importantly, MMP expression associated with chlamydial infection has been shown to be sensitive to IL-17 signaling, with *il17* ^-/-^ mice displaying reduced oviduct expression of MMP2/MMP9 relative to wild-type mice following infection with *C. muridarum* (Andrew et al., 2013). Combined with the observation that IL-17-knockout mice exhibited reduced hydrosalpinx, as well as the consistent association of IL-17 production with genital *C. trachomatis* infection (Jha et al., 2011; Andrew et al., 2013; Masson et al., 2015), it is tempting to speculate that the activity of IL-17-producing cell types may exacerbate *Chlamydia*-associated fibrosis downstream of the host cell response to infection. However, it is critical to note that study of IL-17’s role in fibrosis consistently yields conflicting data (Ramani and Biswas, 2019). Depending on the mode of IL-17 inhibition or knockdown, the cytokine has been shown to either enhance fibrosis by driving fibroblast proliferation and ECM deposition (Chen et al., 2014; Liu et al., 2014), or inhibit fibrosis by antagonizing inflammation and TGFβ-induced myofibroblast activation (Braun et al., 2008; Truchetet et al., 2013). Ultimately, investigation of infection-mediated alterations to ECM architecture may be required to contextualize the effect of MMP induction – and, by extension, the role of IL-17 in *Chlamydia*-associated fibrosis.

Finally, more recent study of the host cell transcriptome in early (1 hpi) and mid-cycle (24 hpi) infection indicates that *C. trachomatis* can induce host expression of fibrillar and fibril-associated collagens (e.g. COL3A1, COL5A1), network-forming collagens (e.g. COL4A1, COL4A2), and laminin/nidogen (e.g. LAMA4, NID1) (Humphrys et al., 2013). The authors additionally observe differential expression of the ADAM (A Disintegrin And Metalloproteinase) and ADAMTS (A Disintegrin And Metalloproteinase with Thrombospondin Motifs) families of ECM remodeling enzymes (Roy et al., 2004; Keating et al., 2006; Janssen et al., 2016), with ADAM33 and ADAMTSL4 repressed relative to mock-infected cells at 1 hour post-infection and ADAM12, ADAM19, ADAMTS3, and ADAMTS6 induced at both 1 and 24 hours post-infection. With the notable exception of membrane-assocated collagen (COL25A1), modulation of collagen expression was largely not detectable at 24 hpi (Humphrys et al., 2013). In combination with the aforementioned reports of increased collagen expression by 48-72 hpi (Porcella et al., 2015; Igietseme et al., 2018), these results imply that *Chlamydia*-mediated induction of a myofibroblast-like phenotype may be required before infected epithelial cells are able to produce collagens. However, secretion of MMP-2 and ADAM/ADAMTS-family proteins may constitute a means by which *Chlamydia* reorganizes the ECM prior to this event, potentially independent of infection-associated EMT. While the field’s understanding of how infection restructures the basement membrane of the upper genital tract is incomplete, further investigation in this area may reveal critical insight into the mechanisms underlying *Chlamydia*-associated fibrosis. Should collagen deposition by *Chlamydia*-induced EMT and infection-associated induction of MMPs/ADAMTS result in a restructured ECM is retained after clearance of the pathogen, the resulting collagen- and matrikine-rich basement membrane of formerly infected tissues may itself act as a pro-fibrotic stimulus. In this way, infection-associated ECM remodeling might be understood as a form of pro-fibrotic imprinting by *Chlamydia*, initiating a program of scar formation that persists between infections.

## 6 Concluding remarks

Considerable progress has been made in the past three decades in understanding how fibrotic pathologies arise from *C. trachomatis* infection. Fibrosis in other contexts is often attributed to chronic inflammation, leading to dysregulation of wound healing and chronic activation and persistence of myofibroblasts, which drive the progressive deposition of ECM components, tissue stiffening, and epithelial cell proliferation associated with scarring (Wynn, 2008; Wynn and Ramalingam, 2012; Distler et al., 2019; Henderson et al., 2020; Tsou et al., 2021). Given that chlamydial infection is consistently associated with pro-inflammatory signaling and immune cell recruitment, chronic inflammation associated with recurrent or persistent infection is thought to be the principal stimulus of scar-forming pathology associated with *Chlamydia*. Initial work posited an immunological paradigm of chlamydial pathogenesis, where chlamydial antigens such as Hsp60 were the principal stimulus of both the host response to infection and the development of fibrotic pathology (Grayston et al., 1985; Arno et al., 1995; Peeling et al., 1997, 60). Further study has dismissed this possibility, however, by discounting the link between chlamydial Hsp60 and the induction of inflammation or fibrosis (Taylor et al., 1987; Yi et al., 1997; Sziller et al., 1998). Subsequent work indicating that *Chlamydia*-infected epithelial cells initiate an extensive pro-inflammatory response capable of recruiting immune cells recommended an alternative hypothesis: that the induction of innate immunity in host cells was the principal driver of the host response to *Chlamydia* (and, thereby, of fibrotic pathology) (Stephens, 2003).

The cellular paradigm of chlamydial pathogenesis offers a compelling explanation for how scarring may arise from the chronic inflammatory stimulus provided by persistent *Chlamydia*, and why repeated infection is associated with higher risk of fibrotic sequelae (Davies et al., 2016), despite no emergent patterns in sensitivity to specific chlamydial antigens. However, this model fails to account for tubal factor infertility and ectopic pregnancy associated with asymptomatic or subclinical infection (Haggerty et al., 2010), as well as the correlation of fibrotic outcomes with induction of humoral immunity (Holland et al., 1993; Bailey et al., 1995; Holland et al., 1996). The incidence of *Chlamydia*-associated scarring in cases of an absent or attenuated response to infection suggests that *C. trachomatis* may act directly as a pro-fibrotic stimulus. *Chlamydia*-infected epithelial cells exhibit multiple phenotypes in support of this hypothesis, including induction of pro-fibrotic cytokines and growth factors (e.g. TGF-β, EGF, VEGF, IL-6, IL-11) (Rasmussen et al., 1997; Coombes and Mahony, 2001; Hess et al., 2001; Dessus-Babus et al., 2002), differential expression of ECM components and maintenance enzymes (e.g. MMP-2, laminins, nidogen) (Ault et al., 2002; Humphrys et al., 2013), and induction of the epithelial-to-mesenchymal transition and associated production of fibrillar collagen (Igietseme et al., 2015; Porcella et al., 2015; Rajić et al., 2017; Igietseme et al., 2018; Zadora et al., 2019; Igietseme et al., 2020). Collectively, these data recommend an expansion to the cellular paradigm of chlamydial pathogenesis: that *Chlamydia* simultaneously induces pro-inflammatory and pro-fibrotic signaling in host epithelial cells, which consequently initiate infection-associated scarring in complementary fashion.

*C. trachomatis* infection demonstrably stimulates fibrosis-associated signaling, promotes expression of ECM components and maintenance enzymes, and induces trans-differentiation of host epithelial cells into collagen-producing myofibroblasts. While much work has been done to characterize the pro-fibrotic phenotypes exhibited by *Chlamydia*-infected epithelial cells, it remains unclear whether these arise solely from the host response to infection, or as a result of pathogen-directed subversion of host cell processes. Past study has indicated that *Chlamydia* can influence host gene expression by manipulating host transcription factors. For example, infection-associated inhibition of the pro-inflammatory transcription factor complex NF-κB has been associated with the chlamydial effector ChlaDub1, which stabilizes the inhibitory subunit IκBα *via* deubiquitination (Le Negrate et al., 2008). Indeed, we have recently reported pathogen-directed induction of the pro-fibrotic transcriptional cofactor YAP during *C. trachomatis* infection of endocervical epithelial cells (Caven et al., 2023). Given the demonstrable role of YAP and other transcription factors in other forms of fibrotic disease (Korol et al., 2016; Gasparics and Sebe, 2018), further study of chlamydial manipulation of host transcription factor activity may be critical to characterizing mechanisms of *Chlamydia*-associated fibrosis.

A critically understudied means through which infection may drive fibrosis is *via* the action of signal factors produced by infected epithelial cells. Infection-associated induction of known inducers of myofibroblast differentiation (e.g. IL-6, IL-8, IL-11, EGF, and CTGF) may facilitate scarring in a paracrine fashion, by inducing myofibroblast differentiation or by sensitizing tissue-resident fibroblasts to pro-fibrotic stimuli. Importantly, myofibroblasts have been consistently identified as the principal mediators of other forms of fibrotic disease (Bochaton-Piallat et al., 2016). Despite this, the role of fibroblast differentiation in *Chlamydia*-associated fibrosis is relatively underexplored – likely given the lack of clinical evidence suggesting fibroblasts are infected by the pathogen *in vivo*. Paracrine signaling by infected epithelial cells may nevertheless constitute a means by which infection induces fibrotic activity in fibroblasts not directly accessible to *Chlamydia*. Indeed, a recent report demonstrated that conditioned media derived from HEK-293 cells expressing constitutively active YAP could induce myofibroblast differentiation *via* the action of the YAP target CTGF (Chen et al., 2020). Indeed, we have recently observed that *C. trachomatis* infection promotes YAP activation *via* a pathogen-directed mechanism involving YAP tyrosine phosphorylation and host Abl kinase activity, with the downstream consequence of YAP- and infection-dependent induction of CTGF expression (Caven et al., 2023). Importantly, YAP activation has been consistently associated with the induction of EMT and fibrosis, *via* its role in mechanotransduction and TGFβ signaling (Szeto et al., 2016; Futakuchi et al., 2018; Park et al., 2019; Mia and Singh, 2022). Taken together, these data suggest that chlamydial YAP activation may stimulate pro-fibrotic activity, both in infected epithelial cells and uninfected cell types *via* the action of CTGF and other YAP-regulated signal factors. Further study of *Chlamydia*-associated fibrosis may thus require assessing the capacity of infected epithelial cells to initiate tissue-level fibrotic activity.

In similar fashion, it is difficult to dismiss potential for infection-associated pro-fibrotic signaling to induce EMT in uninfected, “bystander” epithelial cells. Many pro-fibrotic signal factors expressed by infected epithelial cells have been shown to induce EMT, including EGF, IL-6/8, and CTGF (Palena et al., 2012; Sonnylal et al., 2013; Kim et al., 2016; Abaurrea et al., 2021). *C. trachomatis* infection of murine oviduct epithelial cells additionally induces expression of TGF-β1 *via* engagement with the EGFR receptor during invasion. Critically, pharmacological inhibition of TGF-β and EGFR significantly attenuated infection-associated repression of E-cadherin (Igietseme et al., 2020), suggesting that TGF-β signaling in part drives chlamydial induction of EMT in this cell type. In light of these data, further investigation of the downstream effects of infection-associated pro-fibrotic signaling appears warranted. Further study of pro-fibrotic intercellular communication by host epithelial cells may reveal mechanisms of pathogenesis underlying fibrosis in subclinical and asymptomatic infections *in vivo*, thereby providing novel therapeutic interventions to *Chlamydia*-associated fibrotic disease.

## Author contributions

Conceptualization, LC and RC. Writing – original draft preparation, LC. Writing – review and editing, RC. Supervision, RC. Project Administration, RC. Funding Acquisition, RC. All authors contributed to the article and approved the submitted version.
